# Cross-talk signaling in the trigeminal ganglion: role of neuropeptides and other mediators

**DOI:** 10.1007/s00702-020-02161-7

**Published:** 2020-02-22

**Authors:** Karl Messlinger, Louis K. Balcziak, Andrew F. Russo

**Affiliations:** 1 Institute of Physiology and Pathophysiology, Friedrich-Alexander-University Erlangen-Nürnberg, Universitätsstr. 17, 91054 Erlangen, Germany; 2 Neuroscience Graduate Program, University of Iowa, Iowa City, IA 52242 USA; 3 Department of Molecular Physiology and Biophysics, University of Iowa, Iowa City, IA 52242 USA; 4 Iowa VA Health Care System, Iowa City, IA 52246 USA

**Keywords:** Trigeminal ganglion, Dorsal root ganglion, Sensory neurons, Satellite glial cells, Neuropeptides, CGRP, Signaling

## Abstract

The trigeminal ganglion with its three trigeminal nerve tracts consists mainly of clusters of sensory neurons with their peripheral and central processes. Most neurons are surrounded by satellite glial cells and the axons are wrapped by myelinating and non-myelinating Schwann cells. Trigeminal neurons express various neuropeptides, most notably, calcitonin gene-related peptide (CGRP), substance P, and pituitary adenylate cyclase-activating polypeptide (PACAP). Two types of CGRP receptors are expressed in neurons and satellite glia. A variety of other signal molecules like ATP, nitric oxide, cytokines, and neurotrophic factors are released from trigeminal ganglion neurons and signal to neighboring neurons or satellite glial cells, which can signal back to neurons with same or other mediators. This potential cross-talk of signals involves intracellular mechanisms, including gene expression, that can modulate mediators of sensory information, such as neuropeptides, receptors, and neurotrophic factors. From the ganglia cell bodies, which are outside the blood–brain barrier, the mediators are further distributed to peripheral sites and/or to the spinal trigeminal nucleus in the brainstem, where they can affect neural transmission. A major question is how the sensory neurons in the trigeminal ganglion differ from those in the dorsal root ganglion. Despite their functional overlap, there are distinct differences in their ontogeny, gene expression, signaling pathways, and responses to anti-migraine drugs. Consequently, drugs that modulate cross-talk in the trigeminal ganglion can modulate both peripheral and central sensitization, which may potentially be distinct from sensitization mediated in the dorsal root ganglion.

## Overview of the trigeminal ganglion

**Fig. 1:**
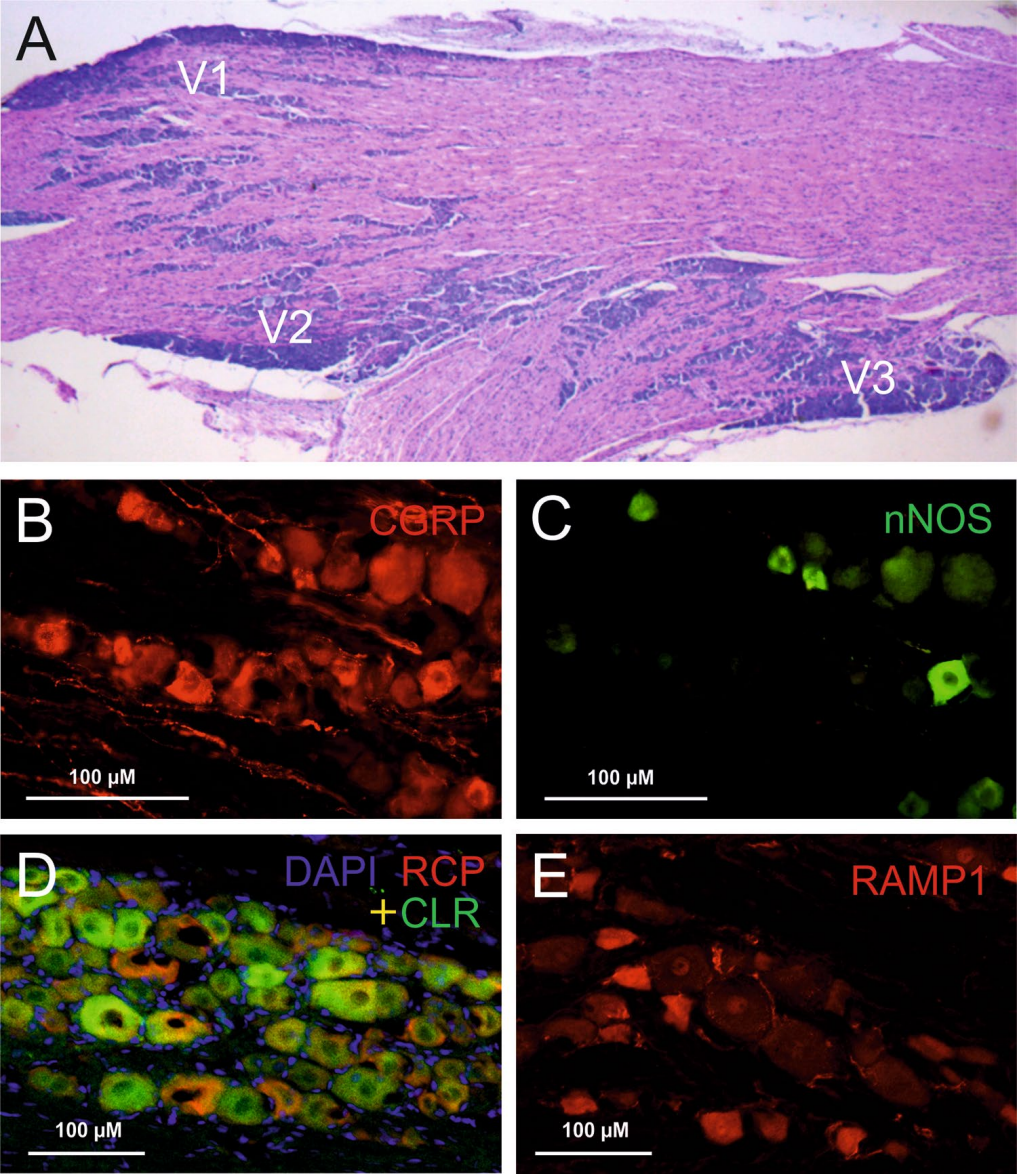
Histology and immunohistochemistry of rat trigeminal ganglion. **a** Horizontal section (hematoxylin–eosin staining) showing clusters of primary afferent somata (dark violet) in the ophthalmic (V1), maxillary (V2) and mandibular division (V3). **b–e** are from the V1 region. **b**,** c** Neurons showing immunofluorescence for CGRP (red) and neuronal NO synthase (nNOS, green), respectively, in the same section. Of the 4 large, about 10 medium-sized and about 15 small neurons, 3 medium-sized and 7 small neurons are clearly CGRP immunopositive, and 4 neurons are also immunopositive for nNOS. Courtesy of Anne Dieterle, Erlangen. **d** Double immunostaining for the CGRP receptor components RCP (red) and CLR (green) plus DAPI nucleus staining (blue) indicating functional units. Several neurons show both RCP and CLR immunoreactivity (yellow). **e** Neurons immunostained for the CGRP receptor components RAMP1 (red). RAMP1 immunoreactive neurons are generally less frequent than CLR and RCP immunoreactive neurons. Courtesy of Mária Dux, Szeged

The trigeminal ganglion is outside the blood–brain barrier (Eftekhari et al. 2015b), which allows substances such as neuropeptides released in the trigeminal ganglion to enter the circulation. The trigeminal ganglion gives rise to three large cranial nerves containing mainly the peripheral axons of pseudo-unipolar primary afferent neurons, the ophthalmic (V1), the maxillary (V2), and the mandibular (V3) nerves; in rodents the latter two form one thick bundle at their origin (Fig. 1a). Results from a retrograde labeling study provided some of the first evidences of cross-excitation from V3 to V1 and V2 within the ganglion, including communication between neurons and satellite glial cells (SGCs) discussed below (Thalakoti et al. 2007; Durham and Garrett 2010; Spray et al. 2019). The central processes of the trigeminal afferents arise from the trigeminal ganglion forming the trigeminal nerve that enters the brainstem at the pontine level. While our focus is on the sensory cell bodies and fibers, there is also a bundle of trigeminal motor fibers, with their somata in the midbrain, that runs through the trigeminal ganglion (Young and Stevens 1979). The trigeminal ganglion consists mainly of primary afferent neurons of the pseudo-unipolar type and glial cells. In human trigeminal ganglion, 20–35,000 neurons and about 100 times more non-neuronal cells have been counted (LaGuardia et al. 2000). The neurons can unequivocally be identified by their nearly round and centrally located nucleus, in which nucleoli and chromatin particle may be visible (Wu et al. 2013). In the rat, the diameters of neurons range from about 10 to 60 µm, with more than 90% small to medium-sized neurons measuring 15–35 µm in diameter (Ambalavanar and Morris 1992; Lennerz et al. 2008). The cell bodies are frequently surrounded by a more or less tight single layer of SGCs that may form a functional unit with the neurons signaling one to the other (Durham and Garrett 2010). In the embryonic trigeminal ganglion, each neuroblast is already accompanied by 2–4 glial cells (Bruska and Woźniak 1991) but the number of SGCs increases by about 20 times in the adult rat trigeminal ganglion, concomitant with the increase in inwardly rectifying potassium channels (Kir4.1), the vesicle docking protein SNAP-25 and the neuropeptide CGRP (Durham and Garrett 2010). The distal and central processes are wrapped by Schwann cells, which form a myelin sheath in Aβ and Aδ fiber neurons. Nonmyelinating Schwann cells are found around processes of C fibers. Surrounding the nerves and in the ganglia are fibroblasts forming collagen fibers, small blood vessels (mainly capillaries) and several types of immune cells such as resident microglia-like macrophages (Glenn et al. 1993). A functional cross-talk between neurons and macrophages via purinergic P2X3 receptors and/or SGCs via P2Y receptors is assumed at least in pathological states like in temporomandibular inflammation (Franceschini et al. 2012; Villa et al. 2010).

## Neuropeptide-producing neurons in the trigeminal ganglion

Trigeminal ganglion neurons express a wide range of neuropeptides. The neuropeptides calcitonin gene-related peptide (CGRP) and substance P were first immunohistochemically described in dorsal root ganglion neurons, with broad overlap in shared secretory vesicles but double the number of vesicles with CGRP immunoreactivity (Wiesenfeld-Hallin et al. 1984). Shortly thereafter CGRP and substance P were localized by immunohistochemistry in trigeminal ganglia of different species (Lee et al. 1985; Hanko et al. 1986; Edvinsson et al. 1989). Trigeminal neuropeptides are still of prominent interest due to their involvement in the generation of primary headaches like migraine and cluster headache (Riesco et al. 2017; Tajti et al. 2015; Ashina et al. 2018). Immunostaining studies have revealed several additional neuropeptides, such as neurokinin A (NK-A), cholecystokinin (CCK), galanin (GAL), somatostatin (SOM), and opioid peptides, in the trigeminal ganglion, which have been comprehensively reviewed (Lazarov 2002; Tajti et al. 2015). Most of the peptide-expressing neurons are small or medium-sized and are thus likely nociceptive neurons with Aδ and C fibers that innervate intracranial structures like the dura mater and cerebral arterial vessels (Mayberg et al. 1984; Schueler et al. 2014). According to more recent data from immunostaining and RT-PCR, further neuropeptides, which can be co-localized with CGRP or substance P, such as enkephalins (Quartu and Del Fiacco 1994), nociceptin (Hou et al. 2003), the growth-associated protein GAP-43 (Del Fiacco et al. 1994), pituitary adenylate-cyclase activating polypeptide (PACAP) (Jansen-Olesen et al. 2014) and angiotensin II (Imboden et al. 2009) are present in the trigeminal ganglion. The ratio of neurons found to produce CGRP and substance P seems to depend on the species and differences in staining, however, there is agreement that CGRP is the most prominent neuropeptide with about 40–50% of CGRP-immunoreactive neurons (Lennerz et al. 2008; Eftekhari et al. 2010) (see Fig. 1b). Regarding the role of substance P, it should be noted that the source of substance P from nociceptors is mainly based on non-human experiments. This may help explain the failure of NK1 receptor antagonists for migraine treatment, whereby substance P release is minor, NK1 receptors in the human meninges that cause plasma extravasation may be lacking, and plasma extravasation, if it occurs, cannot activate nociceptors. As further discussed in the last section, the proportion of CGRP-positive neurons is in the same range as in dorsal root ganglia (Kestell et al. 2015), however, in neurons innervating intracranial blood vessels, CGRP has been found enriched compared to neurons innervating facial skin (O’Connor and van der Kooy 1988). This may be a general principle regarding visceral afferent innervation (Horgan and van der Kooy 1992).

Nociceptive afferents are traditionally grouped into two different populations according to their expression of vanilloid-sensitive transient receptor potential (TRPV1) channels or their isolectin B4 (IB4) binding. The first group containing the peptidergic neurons is sensitive to nerve growth factor (NGF), while the second group is sensitive to glial cell line-derived neurotrophic factor (GDNF) during its development (Vedder et al. 1993; Price et al. 2005). This separation seems not very clear cut, because considerable co-localization of TRPV1 and IB4 binding in primary afferent neurons of rat and mouse, particularly in the rat trigeminal ganglion, has been described based on immunostaining (Price and Flores 2007). Interestingly, in this paper 70% of CGRP-immunoreactive neurons have been found colocalized with TRPV1 immunoreactivity. This was significantly more compared to the dorsal root ganglia and may be one explanation for the dominant role of CGRP as a signaling neuropeptide in the trigeminal system.

Functionally, CGRP release from isolated trigeminal ganglion or trigeminal ganglion cell cultures stimulated by noxious irritants is used as a measure for mass activation of trigeminal ganglion neurons (Durham and Russo 1999; Mason et al. 1984; Bellamy et al. 2006; Eberhardt et al. 2008; Kageneck et al. 2014), keeping in mind that this signal is predictive only for the peptidergic fraction of neurons. Release experiments from the intact trigeminal ganglion using microdialysis is restricted to smaller molecules like substance P (Neubert et al. 2002), because the CGRP molecule is too large for passing microdialysis membranes. The effective role of neuropeptides like CGRP and substance P as signaling molecules is well established in peripheral tissues. Assuming that neuropeptides can also provide intercellular signaling in the trigeminal ganglion, the existence of neuropeptide receptors is an essential prerequisite.

## Neuropeptide receptors in the trigeminal ganglion

CGRP receptors are heteromers, composed of the calcitonin receptor-like receptor (CLR), a seven transmembrane spanning protein, the receptor activity-modifying protein 1 (RAMP1), a single membrane-spanning protein, and the receptor component protein (RCP) an intracellular component (Fig. 1d, e). RAMP proteins are required for the trafficking of CLR from the endoplasmic reticulum to the plasma membrane, and specific RAMPs define the ligand specificity of the calcitonin receptor family (McLatchie et al. 1998). CLR combined with RAMP2 or RAMP3 forms receptors with high affinity for adrenomedullin, another peptide of the calcitonin family (Hendrikse et al. 2019). On the other hand, if RAMP1 is combined with the calcitonin receptor instead of CLR, the amylin-1 receptor is formed, which is discussed below.

In the trigeminal ganglion of several species, neurons of mainly medium sizes and glial cells (Schwann cells and SGCs) have been found to be immunopositive for both CLR and RAMP1 (Lennerz et al. 2008; Eftekhari et al. 2015a) (Fig. 1d, e). There is virtually no overlap of CGRP and CGRP receptor expression, separating the ganglion neurons into CGRP-secreting and a CGRP-responding fractions (Lennerz et al. 2008; Eftekhari et al. 2016). An antibody specifically recognizing a fusion protein of the extracellular domains of RAMP1 and CLR, which comprise the CGRP binding pocket, was used to identify the distribution of CGRP receptors in the trigeminal ganglion of monkey and man. The study confirmed the location of CGRP receptors on neurons and SGCs (Miller et al. 2016).

In addition to the canonical CLR/RAMP1 CGRP receptor, the presence of a second CGRP receptor in the trigeminal ganglion, comprised of the calcitonin receptor (CTR) and RAMP1, is now established (Walker et al. 2015; Hendrikse et al. 2019). The CTR/RAMP1 complex was originally identified as an amylin receptor, hence it is called the AMY1 receptor. Interestingly, the expression pattern of AMY1 receptor is suggestive of co-localization with CGRP, as discussed below, although this remains to be demonstrated. AMY1 receptors are also present in vascular smooth muscle based on immunostaining (Walker et al. 2015) and suggested by functional data from cell culture studies (Bohn et al. 2017). CGRP receptor expression in the trigeminal ganglion is possibly involved in signaling mechanisms that may be important for sensitizing mechanisms in facial pain and headache generation. A possible site of action within the trigeminal ganglion is discussed below.

The existence of neurokinin-1 (NK1) receptors, receptors for substance P, has indirectly been shown by functional studies in the rat trigeminal ganglion. The activity of spinal trigeminal neurons with afferent input from inflamed temporomandibular joint and facial skin was decreased by injection of an NK1 receptor antagonist into the trigeminal ganglion (Takeda et al. 2012). However, it is not likely that NK1 receptors in the trigeminal ganglion are crucially involved in the generation of migraine pain, since NK1 receptor antagonists are ineffective in migraine therapy or prevention of migraine (Goldstein et al. 1997, 2001). PACAP binds to the three receptor subtypes of the VIP/PACAP receptor family, VPAC1, VPAC2 and PAC1, immunoreactivity of which was found in small diameter neurons in rat and human trigeminal ganglion (Chaudhary and Baumann 2002; Knutsson and Edvinsson 2002). The receptors are coupled to Gs-proteins inducing the same intracellular pathways as CGRP, PAC1 is additionally Gq-protein coupled (Rubio-Beltrán et al. 2018). Release of PACAP within the trigeminal ganglion could thus initiate communication between neighboring trigeminal sensory neurons.

Evidence for neuropeptide receptors that are linked to inhibitory Gi-proteins has also been found in by immunohistochemistry and in situ hybridization in the trigeminal ganglion. The presence of somatostatin receptors (sst2A) (Ichikawa et al. 2003) and galanin receptors (GALR1) (Suzuki et al. 2002) has thus been shown in small- to medium-sized neurons in the rat trigeminal ganglion. Binding sites for cholecystokinin (CCK) have also been localized in the trigeminal ganglion of different species (Ghilardi et al. 1992). In addition, delta opioid receptor binding sites were observed (Ichikawa et al. 2005) and were upregulated following experimental inflammation of the tooth pulp (Huang et al. 2015). It is unknown if these receptors, which are usually linked to an antinociceptive function, have a local inhibitory role in the trigeminal ganglion.

Other neuropeptides, which may have a signaling function in the trigeminal ganglion, but originate most likely from other sources, are orexins and oxytocin. Orexin receptor (OX_1_R and OX_2_R) mRNA has been detected in rat trigeminal ganglion neurons and inhibition of both receptors reduced the expression of downstream proteins associated with sensitization of peripheral nociception in a model of temporomandibular joint inflammation (Cady et al. 2014). Oxytocin receptor immunoreactivity has also been found in rat trigeminal neurons, the majority of which also co-expressed CGRP (Tzabazis et al. 2016). In a recent study oxytocin suppressed neuronal hyperexcitability of trigeminal ganglion neurons after nerve injury. This was mediated by modulation of K^+^ channels through activation of vasopressin-1-receptors, immunoreactivity for which has also been found in trigeminal ganglion neurons (Kubo et al. 2017).

## Receptors for other signal molecules expressed in the trigeminal ganglion

In addition to the variety of neuropeptide receptors in the trigeminal ganglion, multiple receptors for neurotrophic factors and other receptors involved in sensory transduction and transmission have been found expressed on the mRNA level or by immunohistochemistry (Lazarov 2002) or have been found by classical pharmacological approaches. Frequently, cultured trigeminal ganglion cells have been used for studies on sensory transduction or presynaptic mechanisms of neurotransmission, for which the cell soma is used as a model of its peripheral or central ending, respectively. Functional receptors expressed in the ganglion may be involved in intercellular signaling, as will be discussed below.

Receptors for classical neurotransmitters are abundantly expressed in the trigeminal ganglion. Using immunohistochemistry, receptor proteins for all types of glutamate receptors, AMPA, kainite, N-methyl-D-aspartate (NMDA) and metabotropic glutamate receptors (mGluR), have been localized in rat trigeminal ganglion neurons (Quartu et al. 2002; Yang et al. 2009; Sahara et al. 1997), and mGluR proteins have also been found in SGCs (Boye Larsen et al. 2014). Besides their role in neurotransmission, NMDA receptors may functionally interact with transient receptor potential (TRPV1) receptor channels contributing to mechanical hyperalgesia (Lee et al. 2012).

Expression of subunits of nicotinergic (nAchR) and muscarinic acetylcholine receptors (mAchR) has been found on the mRNA level and with immunohistochemistry in rat trigeminal ganglion (Flores et al. 1996; Dussor et al. 2004). Considerable proportions of cultured trigeminal ganglion neurons responding to carbachol and nicotine with calcium transients were found suggesting that both nAchR and mAchR are functional (Shelukhina et al. 2017). This may be important for the idea that parasympathetic nerve fibers signal to trigeminal afferents possibly promoting trigeminal autonomic cephalalgias and migraine characterized by autonomic symptoms (Goadsby 2005; Barbanti et al. 2016).

About 70% of rat trigeminal ganglion neurons have been found to be GABAergic by immunohistochemistry, with various subtypes of GABA receptor subunits identified by RT-PCR and in situ hybridization (Hayasaki et al. 2006). GABA was released by strong depolarizing stimuli (high molecular K^+^ solution) and Cl^−^ currents recorded in whole cell patch clamping showed that the subunits form functional GABA receptors. The authors discussed a possible GABA-driven inhibition of neurons within the trigeminal ganglion. Glycine receptors were also found in rat trigeminal ganglia using immunohistochemistry (Bae et al. 2016).

Serotonin (5-hydroxytryptamine, 5-HT) binds to several types of G-protein-coupled 5-HT receptors, only the 5-HT3 receptor is a cation channel. Three subtypes of the Gi-protein-coupled serotonin 1 receptors are targets of antimigraine triptans (5HT1B/1D) or “ditans” like lasmitidan (5HT1F) and have been found by immunohistochemistry in the rat trigeminal ganglion (Classey et al. 2010). Interestingly, there was no difference in receptor density compared to dorsal root ganglia showing that the 5HT1 receptor equipment is not specific for the trigeminal system. However, one caveat is that immunohistochemistry does not necessarily reflect functional receptors. For example, the 5HT1D receptor is held in internal stores and only translocated to the cell surface of dorsal root ganglia neurons following neural stimulation (Ahn and Basbaum 2006). In human trigeminal ganglia, 5-HT1B and 5-HT1D receptor immunoreactivity was found predominantly in medium-sized neurons, colocalized with CGRP, substance P or nitric oxide synthase, confirming a close association of 5-HT1 activation and inhibition of neuropeptide release (Hou et al. 2001).

Purinergic receptors binding ATP and other purines are either G-protein coupled (P2Y) or form cation channels (P2X). Expression of different subtypes of P2X receptors, predominantly P2X2 and P2X3, was described in rat trigeminal ganglion neurons of small and medium size, frequently co-expressed with neuropeptides (Xiang et al. 1998; Staikopoulos et al. 2007; Ambalavanar et al. 2005). P2X3 receptor expression in cultured trigeminal ganglion neurons has been found to be enhanced by CGRP and nerve growth factor (Giniatullin et al. 2008; Simonetti et al. 2008) and functionally downregulated by brain natriuretic peptide (Marchenkova et al. 2015). P2X3 receptors may be involved in trigeminal neuropathic and inflammatory pain (Shinoda et al. 2007; Teixeira et al. 2010). Immunohistochemical and functional data suggest that P2Y receptors are not expressed by neurons but rather by glial cells in rodent trigeminal ganglia (Weick et al. 2003). Cell cultures imply a bidirectional signaling between neurons and glia cells via ATP (Suadicani et al. 2010), which seems to be enhanced in Ca(v)2.1 α1 R192Q mutant knock-in mice as a model of familial hemiplegic migraine type 1 (Ceruti et al. 2011). Trigeminal ganglion neurons can release ATP upon noxious chemical stimulation (Neubert et al. 2002) and may thus be involved in purinergic signaling within the ganglion, as discussed later.

Significant proportions of trigeminal ganglion neurons express receptors of the transient receptor potential (TRP) family. TRP receptors form transduction channels in peripheral sensory endings and may also be involved in synaptic transmission at the central afferent terminals (Raisinghani et al. 2011). Immunoreactivity for the TRP vanilloid type 1 receptor channel (TRPV1) was found colocalized with CGRP in most of the trigeminal ganglion neurons (Hou et al. 2001). This nonspecific cation channel can be activated by exogenous substances like capsaicin or resiniferatoxin, noxious heat, acidic pH (pH < 5.3), and different endogenous compounds including membrane-derived lipid metabolites like anandamide (Price et al. 2004). CGRP release from trigeminal ganglia or trigeminal ganglion cell cultures induced by capsaicin is frequently used as a measure for trigeminal activation (Thalakoti et al. 2007; Meng et al. 2009). Another member of the TRP receptor family, the transient receptor potential ankyrin 1 (TRPA1) channel, which is highly colocalized with TRPV1 receptors in trigeminal neurons, is activated by irritating substances like mustard oil and cannabinoids (Salas et al. 2009; Jordt et al. 2004). This receptor channel can also be activated by volatile constituents such as umbellulone of the “headache tree” (Nassini et al. 2012). Its functional role in trigeminal nociception is controversial, because on one hand there is experimental evidence for a cooperative effects with TRPV1 in meningeal afferents (Denner et al. 2016) but on the other hand for a dual nociceptive–antinociceptive effect when recordings were made from spinal trigeminal neurons (Teicher et al. 2017). In addition, TRP channels of the M8 type (TRPM8) are expressed in trigeminal ganglion neurons. The transduction channel TRPM8 is interesting, because genome-wide association studies showed that it may be implicated in migraine (Chasman et al. 2011). In a rat behavioral model of headache, the TRPM8 agonist icilin applied onto the cranial dura mater was shown to produce cutaneous facial and hind paw allodynia that was attenuated by systemic pretreatment with a TRPM8 antagonist (Burgos-Vega et al. 2016). On the contrary, TRPM8 activation reversed meningeal inflammation-induced lowering of the facial heat pain threshold, and in a trigeminal ganglion cell assay TRPM8 activation inhibited TRPV1 effects, which raised speculations about an antinociceptive activity of TRPM8 in migraine (Kayama et al. 2017). Thus, it seems that TRPM8 activation by exogenous agonists can both aggravate and alleviate headache-related behaviors, possibly depending on stimulation of other pro-nociceptive receptors of meningeal afferents (Dussor and Cao 2016).

Other proton-activated transduction channels identified in trigeminal ganglion neurons are the acid-sensing ion channels (ASICs), predominantly the ASIC3 subtype, which is suggested to contribute to headaches under acidic or inflammatory conditions (Yan et al. 2013). Acidic metabolites released under ischemia as a consequence of cortical spreading depression during the aura phase of migraine have been speculated to contribute to the generation of migraine pain (Dussor 2015). Interestingly, CGRP release from cultured trigeminal ganglion neurons induced by protons (pH 5.5) was blocked by an ASIC3 inhibitor but not by the calcium-binding complex EGTA or the antimigraine drugs onabotulinum toxin A or rizatriptan, suggesting that the H^+^-evoked CGRP release upon ASIC3 opening is not (alone) controlled by calcium binding proteins usually involved in vesicular exocytosis (Durham and Masterson 2013).

## Intercellular cross-talk within the trigeminal ganglion

Multiple findings regarding the release of chemical signals from trigeminal ganglion cells, cellular responses to these signals and intracellular mechanisms suggest that an intense cross-talk between different cell types may take place within the ganglion. An important way of communication between neurons and satellite glial cells seems to be established via gap junctions, evidence for which was reported already more than 20 years ago (Thalakoti et al. 2007). Capsaicin injected into the temporomandibular joint induced spreading of True Blue between neuronal cell bodies and adjacent glial cells, concomitant with increased expression of inflammatory proteins in both neurons and glia. Injection of the inflammatory cytokine TNFα and capsaicin into rat facial skin was followed by increased expression of the gap-junction forming protein connexin 26 in trigeminal ganglion neurons and SGCs (Damodaram et al. 2009). Similarly, after inferior alveolar nerve injury mechanical allodynia in the whisker pad was accompanied by an enhanced expression of connexin 43 in trigeminal ganglion SGCs, suggesting that glia activation has significant impact on the excitability of nociceptive trigeminal ganglion neurons (Kaji et al. 2016). Functional gap junctions between cultured mouse trigeminal ganglion neurons and glial cells have also been substantiated by recording bidirectional electrical responses with patch clamp techniques (Spray et al. 2019).

**Fig. 2:**
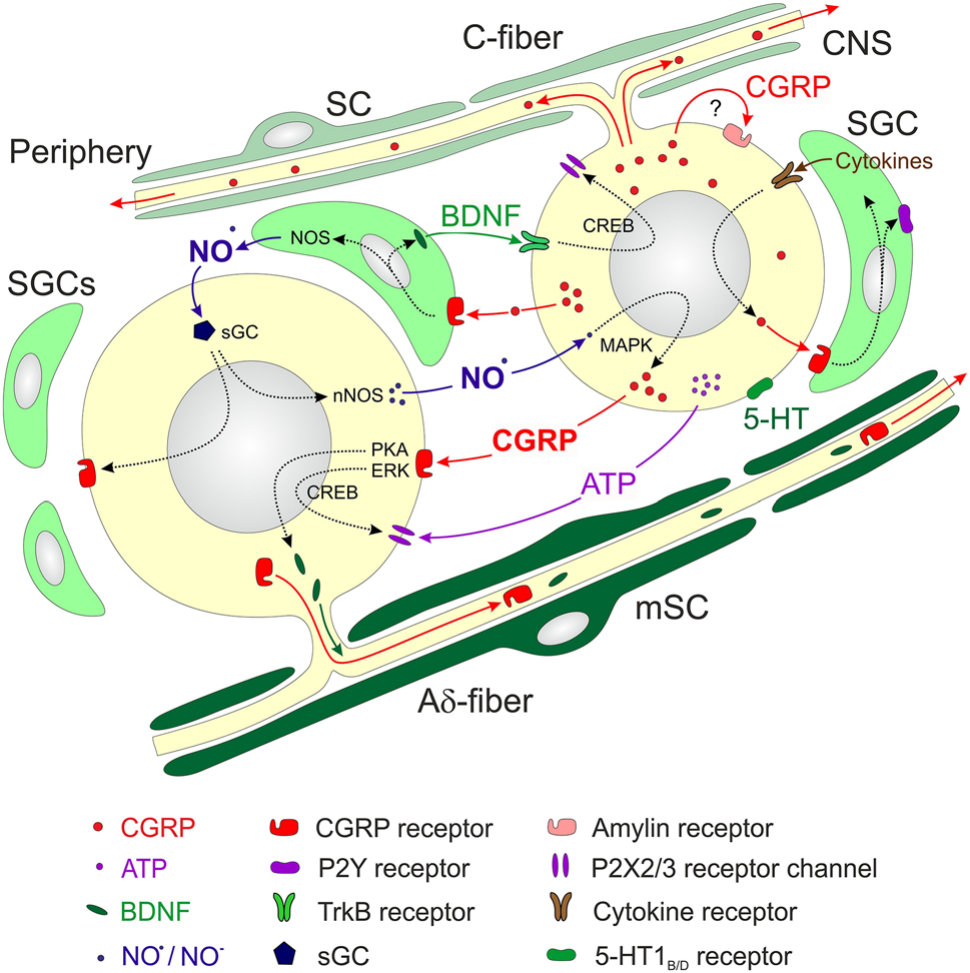
Representation of receptor expression and signaling processes in and between trigeminal ganglion cells. Small neurons (with C-fibers) expressing CGRP may signal to satellite glial cells (SGCs) and to middle-sized neurons (with Aδ or C-fibers) expressing CGRP receptors. CGRP release by Ca^2+^-dependent exocytosis can be induced by activating Ca^2+^-conducting ion channels like TRPA1, for example, by nitroxyl (NO^−^). Autocrine activation by CGRP may occur via CGRP-binding amylin receptors. CGRP and amylin receptors may activate intracellular cascades involving cAMP response-element binding protein (CREB) or mitogen-activated protein kinase (MAPK) to induce gene expression of purinergic (P2X3) receptor channels in neurons and purinergic (P2Y) receptors in SGCs, enzymes like nitric oxide synthase (NOS), cytokines like tumor necrosis factor (TNFα) as well as growth factors like brain-derived neurotrophic factor (BDNF). Nitric oxide (NO), cytokines and BDNF may signal back to neurons facilitating expression of purinergic receptor channels, CGRP and CGRP receptor components like RAMP1. In addition, ATP released from neurons may activate SGCs and macrophage-like cells (MLC), which can signal back to neurons by cytokines. Many of the gene products like CGRP, CGRP receptor proteins and BDNF can crucially influence neuronal transduction and synaptic transmission, because they are delivered by axonal transport through the neuronal processes to the peripheral and/or central terminals of trigeminal afferents

Apart from these direct communication mechanisms, intercellular cross-talk by several mediators released from neurons and SGCs seems to be established. In terms of trigeminal nociception and headache generation, the most relevant possible interplay between trigeminal ganglion cells involves CGRP, which may signal to other neurons as well as SGCs and Schwann cells (Fig. 2). This interaction may stimulate various intracellular metabolic changes through the activation of gene expression (Russell et al. 2014). In dorsal root ganglion cell cultures, immunohistochemical data showed that CGRP, via cAMP increase, can cause phosphorylation of cAMP response element binding (CREB) protein, suggesting that CGRP can regulate its own gene expression by pathways involving protein kinase A and mitogen-activated protein kinase/extracellularly regulated kinase (ERK) (Anderson and Seybold 2004). Similar signaling mechanisms have been demonstrated in the trigeminal system (Zhang et al. 2007; Walker et al. 2017). Autocrine regulation of CGRP transcription has been speculated to occur in cerebellar Purkinje neurons (Edvinsson et al. 2011). While colocalization of CGRP and CGRP receptor elements was very rarely seen in trigeminal ganglion neurons (Eftekhari et al. 2010; Lennerz et al. 2008; Tajti et al. 1999), autocrine regulation cannot be ruled out because of the discovery of a second CGRP receptor and the reported plasticity of RAMP1 expression. With respect to the second receptor, as mentioned earlier, the AMY1 receptor is localized primarily in small and medium diameter trigeminal ganglion neurons (Walker et al. 2015), which is in contrast to localization of the CLR/RAMP1 receptor primarily in larger diameter neurons (Eftekhari et al. 2010; Lennerz et al. 2008).

Apart from autocrine functions, CGRP released from trigeminal ganglion neurons can stimulate surrounding cells leading to an enhancement of ATP-gated purinergic P2Y receptors in SGCs (Ceruti et al. 2011) and P2X3 receptors in other neurons (Giniatullin et al. 2008; Simonetti et al. 2008). Control of the purinergic P2X3 receptor by CGRP is of particular interest since it involves two mechanisms (Simonetti et al. 2008), which could come into play during migraine (Fig. 2). First, CGRP can directly act on neurons to initiate a cAMP-signaling cascade that activates the P2X3 gene. Second, CGRP can indirectly act via activation of the neurotrophin BDNF gene and BDNF release from SGCs, which can then upregulate P2X3 expression in neurons. Like CGRP, BDNF is elevated during migraine (Fischer et al. 2012), suggesting that these two mediators act together in augmenting purinergic receptors in migraine. Whether BDNF or P2X3 receptors then feedback to increase CGRP synthesis is not known, but it seems likely, given that they activate pathways known to increase CGRP gene transcription (Durham and Russo 2003; Viney et al. 2004; Zhang et al. 2007). The end result of this interplay would be to promote depolarization of trigeminal afferents and transmission of nociceptive stimuli (Souslova et al. 2000). Furthermore, dynamic regulation of CGRP receptor subunits by other migraine-relevant stimuli (e.g., stress and hypoxia) has also been reported (Hay et al. 2006). Thus, there is the possibility of increased CGRP synthesis in response to migraine triggers via paracrine- and autocrine-positive feedback loops.

CGRP released from neurons is also suggested to stimulate the expression and release of nitric oxide (NO) and cytokines in SGCs (Li et al. 2008; Thalakoti et al. 2007; Vause and Durham 2010) (Fig. 2). Treatment of primary trigeminal ganglia cultures with CGRP increased the level of multiple cytokines, including TNF-α and IL-1β (Thalakoti et al. 2007; Vause and Durham 2010). CGRP was shown to enhance the production of interleukin 1β (IL-1β) in SGCs, while trigeminal ganglion neurons produced nitric oxide (NO) (Capuano et al. 2009). Conversely, the cytokine TNF-α was shown to increase CGRP gene transcription and peptide secretion (Bowen et al. 2006). Likewise, medium from SGCs activated by either IL-1β or NO augmented the evoked release of CGRP from trigeminal neurons. There is evidence that the CGRP releasing effect of NO donors on trigeminal ganglion neurons is caused by activation of the CGRP promoter activity, which was partly suppressed by the antimigraine compound sumatriptan (Bellamy et al. 2006). Infusion into the rat of glycerol trinitrate (GTN), which mimics NO in activating soluble guanylate cyclase (sGC) (Kleschyov et al. 2003), was followed by an increase in CGRP and nNOS immunoreactive neurons (Dieterle et al. 2011). The same treatment also increased the number of neurons immunoreactive for RAMP1, the rate-limiting component of the CGRP receptor, while sGC immunoreactivity in trigeminal ganglion neurons was decreased (Seiler et al. 2013). In addition to NO, the gaseous transmitter H_2_S, which together with NO forms nitrosyl (NO^−^), a TRPA1 receptor activating sibling of NO, may play a similar role in the ganglia by increasing the release of CGRP (Eberhardt et al. 2014; Dux et al. 2015). In this context, a recent paper reported that facial allodynia in mice depends on functional TRPA1 receptor channels and is associated with signs of oxidative stress in trigeminal ganglion neurons (Marone et al. 2018).

In summary, these observations indicate that CGRP could function as a paracrine factor to stimulate nearby glial cells and neurons, which in turn could feedback with signal molecules like NO to further stimulate CGRP synthesis and release. This would generate a perfect scenario of a vicious circle for sensitizing trigeminal ganglion neurons, which may contribute to pain exacerbation, as in migraine. However, it should be emphasized that most of the findings mentioned are from cell cultures, where the natural composition and architecture of the ganglion is lost, and therefore the discussed data are suggestive of possible mechanisms that need to be confirmed in vivo. In that regard, substance P release has been reported not only from cultured trigeminal ganglion neurons (Wang et al. 2016) but also from intact trigeminal ganglia using microdialysis (Neubert et al. 2002). Using this method for measuring CGRP release is limited by the higher molecular weight of this neuropeptide that does not readily pass the microdialysis membranes with sufficient selectivity.

Other signaling mechanisms between primary afferent neurons, SGCs and immune cells do not directly involve neuropeptides and NO. Intraganglionic signaling by ATP has been reviewed elsewhere (Goto et al. 2017). Neurons may signal via ATP to other neurons and to SGCs, and these may signal to microglia/macrophage-like cells (MLCs). SGCs and MLCs may signal back to neurons via cytokines and neurotrophic factors thus inducing a cross-talk of sensitization. Likewise, glutamate can be a neuro-glial transmitter within sensory ganglia. In rat trigeminal ganglia KCl stimulation released glutamate when glutamate uptake by SGCs was inhibited. Calcium imaging showed that neurons and SGCs respond to AMPA, NMDA, kainate and mGluR agonists, and selective antagonists blocked this response, which is indicative of functional glutamate receptors of all types (Kung et al. 2013). Inflammation by complete Freund’s adjuvant caused expression of MAPK (pERK1/2, pp38) and NF-κB in the trigeminal ganglion involving both neurons and glia, which indicates neuron–glia interactions. Administration of the NMDA-receptor antagonists, kynurenic acid, inhibited these responses indicating the importance of intraganglionic NMDA receptors (Csáti et al. 2015).

## Significance of intraganglionic cross-talk for nociceptive transmission

As mentioned above, data derived from experiments with isolated trigeminal ganglion cells should be interpreted with care when they are used to explain possible mechanisms of sensory transduction or transmission. However, since most of the molecules generated in the cell body are stored in vesicles (Zhao et al. 2011) and delivered by axonal transport to the periphery and/or the central nervous system (Maday et al. 2014), they may be relevant there. CGRP and other neuropeptides are transported to the peripheral terminals, where they are released and induce processes of neurogenic inflammation like arterial dilatation and plasma extravasation (Moskowitz 1993; Williamson et al. 1997). The neuropeptides are also transported to the central terminals, where they act as neuromodulators (Storer et al. 2004; Coste et al. 2008). Different to the neuropeptides, a more or less unidirectional transport can be assumed for CGRP receptor subunits. Confocal immunohistochemistry has shown the RAMP1 receptor subunit co-localized with axonal markers only in the central but not the peripheral processes of rat trigeminal afferents, indicating an unilateral transport into the central terminals (Lennerz et al. 2008), although there are conflicting data regarding this issue (Eftekhari et al. 2013). The CGRP receptor subunits can be integrated into the presynaptic membrane of central trigeminal terminals forming functional presynaptic CGRP receptors. They may be activated by CGRP released from central terminals of other trigeminal afferents to facilitate neurotransmitter release and synaptic transmission (Takhshid et al. 2007). Similarly, a recent immunohistochemical study identified CGRP receptor subunits at nodes of Ranvier of Aδ fibers adjacent to CGRP-containing C fibers in the ganglia and dura (Edvinsson et al. 2019). Thus, there is a possibility of cross-talk between adjacent fibers as well in cell bodies in the ganglia. Another example is brain-derived neurotrophic factor (BDNF), which is expressed in cultured trigeminal neurons dependent on the presence of CGRP (Buldyrev et al. 2006) (Fig. 2). BDNF can be released from central presynaptic terminals and may act on pre- and postsynaptic tyrosine kinase (TrkB) receptors to facilitate nociceptive transmission (Garraway and Huie 2016).

This scenario is most relevant in the light of recent discussions about the sites of action of monoclonal antibodies, which are assumed to act outside the blood–brain barrier inhibiting CGRP signaling and reducing trigeminal functions involved in migraine (Russo 2015), as reviewed elsewhere (Edvinsson 2015) (DosSantos et al. 2014). In this way, substances that act within the trigeminal ganglion (which is outside the blood–brain barrier) contributing to the intraganglionic cross-talk, as discussed above, can have considerable impact on the peripheral and central functions of nociceptive transduction and transmission, and changes in gene expression or transport of molecules can have consequences for peripheral and central functions.

## Comparison of trigeminal and dorsal root ganglia sensory neurons

A fundamental question is how does the trigeminal ganglion differ from the dorsal root ganglion (DRG)? Both ganglia serve similar roles of encoding somatosensory modalities such as pain, touch, temperature and proprioception. However, despite their functional overlap, there are distinct differences in their ontogeny, gene expression, including distribution of CGRP receptors, and responses to antimigraine drugs.

The trigeminal ganglion and the dorsal root ganglia arise from different embryological origins. Sensory neurons in the trigeminal ganglion are from a mixed origin of both cranial neural crest cells and the trigeminal ophthalmic and maxillomandibular placodes (Baker and Bronner-Fraser 2001; Barlow 2002; Pavan and Raible 2012). In contrast, the dorsal root ganglia originate entirely from trunk neural crest progenitor cells. The differentiation potential of neural crest along the rostral–caudal axis (cranial to trunk) is directed in part by expression of HOX homeotic genes and environmental cues, such as morphogens (Le Douarin et al. 2004; Philippidou and Dasen 2013). For the cranial vs trunk choice, the Wnt morphogen directs neural crest cells to a trunk fate of sensory neuron progenitors (Hackland et al. 2019). Wnt also induces expression of homeobox transcription factors that cause rostral–caudal division in the trunk neurons (Holland and Hogan 1988). Interestingly, the embryonic neurons of the dorsal root ganglia and trigeminal ganglion are transcriptionally similar except for a few gene transcripts such as the homeobox genes up to embryonic day 15 (Eng et al. 2007). Differential homeobox expression and activity may partially drive dorsal root ganglion development that allows it to have unique expression patterns compared to the trigeminal ganglion.

In the adult trigeminal and dorsal root ganglion neurons, there are unique patterns of expression that mirror the embryological differences. Using transgenic mice with GFP expression in only sensory cells, neurons in the DRG and trigeminal ganglion were separated from non-neuronal cell types by FACS and analyzed by RNAseq (Lopes et al. 2017). The two different sensory ganglia had almost identical gene expression with the exception of 63 genes. For example, the dorsal root ganglion had homeobox transcripts that were not present in the trigeminal ganglion. Conversely, the trigeminal ganglion had RNAs encoding vasopressin, oxytocin and GABA receptor subunits. A similar RNAseq study focusing on RNAs being actively translated revealed that the trigeminal ganglion has greater expression of genes in the PI3K–mTORC1 pathway, while inhibitors of the pathway were more prominent in the dorsal root ganglion (Megat et al. 2019). Enhanced expression of PI3K–mTORC1 pathway genes in the trigeminal ganglion was also confirmed at the protein level. The enhanced mTOR pathway may help partially explain why trigeminal neurons have different sensory thresholds compared to dorsal root ganglion neurons.

With respect to CGRP signaling in dorsal root and trigeminal ganglion, there are some potential differences in receptor expression, distribution and site of action. Using immunohistochemistry, in adult trigeminal ganglion neurons the CGRP receptor components RAMP1 and CLR were predominantly found in medium-sized cell bodies, presumably with Aδ fibers, whereas CGRP expression was predominantly seen in small neurons with unmyelinated C-fibers (Lennerz et al. 2008; Eftekhari et al. 2010). Thus, trigeminal ganglion neurons have little or no colocalization of CGRP and its receptor subunits. In contrast, small diameter dorsal root ganglion neurons in rats express CGRP and at least low levels of CLR and RAMP1 colocalized in the cell bodies (Cottrell et al. 2012). However, these differences have not been compared in head to head tests and so may reflect differences in tissue extraction, quality of antibodies and immunostaining protocols.

Likewise, CGRP may act at different presynaptic and postsynaptic sites when released from the trigeminal and dorsal root ganglion neurons, although there are conflicting reports. In the dorsal horn, CLR expression was initially found predominantly on cell bodies and dendrites of second-order neurons (Ye et al. 1999). However, subsequent studies found CGRP receptor subunits to be predominantly presynaptic and only on a few cell bodies in the dorsal horn (Marvizón et al. 2007; Eftekhari and Edvinsson 2011; Cottrell et al. 2012). In the spinal trigeminal nucleus, CGRP receptor subunits were initially identified only on fibers from the trigeminal ganglion, which indicated an exclusively presynaptic localization of CGRP receptors. However, a subsequent study using a different antibody that recognizes the CLR/RAMP1 complex found the receptor predominantly on cell bodies and dendrites and on only some axon terminals (Miller et al. 2016). Hence, it remains unresolved whether CGRP acts presynaptically and/or postsynaptically in the spinal dorsal horn and trigeminal nucleus.

Responses to antimigraine medication may also be a point of difference between the two neuron populations. The 5HT1_B/D_ receptors for triptans are present on both trigeminal and dorsal root ganglion neurons, yet to date it appears that triptans are efficacious for only relief of pain initiated in the trigeminal pathway (Ahn and Basbaum 2005). One study showed that intravenous administration of naratriptan was able to prevent responses in the trigeminal nucleus caudalis to noxious stimuli on the dura mater in anesthetized rats (Cumberbatch et al. 1998). In contrast, in the same study, naratriptan did not affect spinal dorsal horn responses to noxious stimuli. Whether the difference is due to the second-order neurons or primary afferents is not known. Another example is a study in which sumatriptan failed to prevent dorsal root ganglion-mediated pain responses in rodents with a variety of tests such as tail flick or the hot pad (Skingle et al. 1990). Finally, a meta-analysis found that antimigraine medications such as anticonvulsants had different effectiveness ratings in pain conditions such as trigeminal neuralgia or pain due to peripheral nerve injury (Jensen 2002). This points to asymmetric traits of the dorsal root ganglia and the trigeminal ganglion for response to treatments.

Overall, while dorsal root and trigeminal ganglion neurons are similar sensors of peripheral stimuli, they have different embryonic origins, transcription patterns, signaling pathways, and responses to antimigraine medications. Differences between the two groups of sensory neurons may provide clues for selective targeting of CGRP and other targets that modulate peripheral and central sensitization in pain states.
